# ClusterGVis: An Advanced Visualization and Clustering Tool for Gene Expression Analysis

**DOI:** 10.1093/gpbjnl/qzag005

**Published:** 2026-01-22

**Authors:** Jun Zhang (张俊), Hongyuan Li (李弘远), Wenjun Tao (陶文君), Jun Zhou (周君)

**Affiliations:** State Key Laboratory of Natural Medicines, China Pharmaceutical University, Nanjing 211198, China; School of Life Science and Technology, China Pharmaceutical University, Nanjing 211198, China; State Key Laboratory of Natural Medicines, China Pharmaceutical University, Nanjing 211198, China; School of Life Science and Technology, China Pharmaceutical University, Nanjing 211198, China; State Key Laboratory of Natural Medicines, China Pharmaceutical University, Nanjing 211198, China; School of Life Science and Technology, China Pharmaceutical University, Nanjing 211198, China; State Key Laboratory of Natural Medicines, China Pharmaceutical University, Nanjing 211198, China; School of Life Science and Technology, China Pharmaceutical University, Nanjing 211198, China; Jiangsu Key Laboratory of Drug Design and Optimization, China Pharmaceutical University, Nanjing 211198, China

**Keywords:** Fuzzy c-means clustering, Gene clustering visualization, Functional annotation, Single-cell RNA sequencing, Bulk RNA sequencing

## Abstract

Both single-cell RNA sequencing and bulk RNA sequencing data provide valuable insights into physiological and pathological processes. The effective interpretation of such data relies on the availability of sophisticated analytical and visualization tools. Here, we introduce ClusterGVis, an advanced bioinformatics software specifically designed to simplify the analysis and visualization of gene expression data. ClusterGVis provides a user-friendly interface that allows researchers to perform fuzzy c-means and k-means clustering on transcriptomic data. It enables researchers to effectively uncover patterns and relationships within complex gene expression profiles. The integrated heatmap visualization features support intuitive exploration of co-expression networks and identification of differentially expressed genes across diverse experimental conditions. ClusterGVis serves a dual purpose: aiding in the identification of potential biomarkers and enriching the understanding of gene function and regulatory mechanisms. The tutorials, manual, source code, and demo data of ClusterGVis are publicly available at https://github.com/junjunlab/ClusterGVis and https://bioconductor.org/packages/ClusterGVis. The ClusterGVis Shiny app has been deployed on shinyapps.io and is accessible at https://laojunjun.shinyapps.io/clustergvis_app_v0/. The Shiny app source code is hosted on GitHub at https://github.com/junjunlab/ClusterGvis-app.

## Graphical abstract

**Figure:**
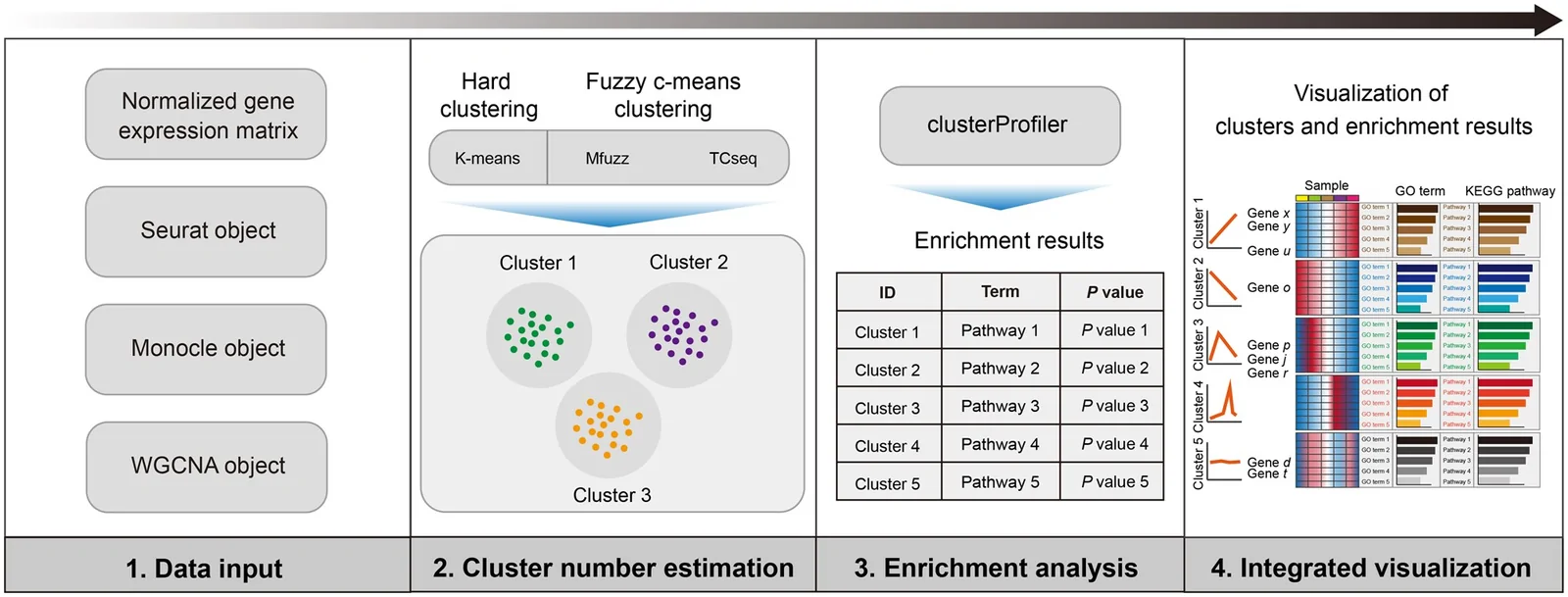

## Introduction

The analysis of gene expression data obtained from high-throughput sequencing is crucial for elucidating biological mechanisms. In the field of genomics, tools for gene clustering, pathway enrichment analysis, and result visualization tend to be fragmented and standalone. An overview of existing tools for these commonly used analyses is provided in **Table 1** [1–3]. Although a variety of R packages for functional enrichment analysis are available (including enrichR [4], GSEA [5], and clusterProfiler [6,7]), there is a notable lack of tools that can classify genes based on their distinct expression patterns and conduct comprehensive batch enrichment analyses. Furthermore, data integration, clustering, visualization, and enrichment result processing can be excessively time-consuming for researchers. For example, despite its broad capabilities, the ComplexHeatmap R package [8] is challenging for beginners. This underscores the need for more integrated and user-friendly tools to simplify genomic data analysis and visualization. In response to this demand, we present ClusterGVis, a robust and user-friendly bioinformatics tool specifically designed for gene expression data clustering and visualization.

**Table 1:** Comparison of ClusterGVis with existing tools

	Characteristic	New	Time-series analysis			Enrichment tool		Visualization	
		ClusterGVis	STEM	Mfuzz	TCseq	clusterProfiler	gprofiler2	ComplexHeatmap	simplifyEnrichment
Software distribution metadata	Version	0.99.9	1.3.13	2.64.0	1.28.0	4.12.6	0.2.3	2.20.0	1.99.0
	Installation repository	Bioconductor, GitHub	Project website	Bioconductor	Bioconductor	Bioconductor, GitHub	CRAN	Bioconductor, GitHub	Bioconductor, GitHub
	Main environment	R (≥ 4.5.0)	Java (≥ 1.4)	R (≥ 2.5.0)	R (≥ 3.4)	R (≥ 3.5.0)	R (≥ 3.5.0)	R (≥ 3.5.0)	R (≥ 4.0.0)
	Freeware license	MIT	GPL-3.0	Apache-2.0	GPL-2.0	Artistic-2.0	GPL-2.0	MIT	MIT
Feature-specific comparison	Clustering analysis	Yes	Yes	Yes	Yes	No	No	No	Yes
	Functional enrichment	Yes	Yes	No	No	Yes	Yes	No	No
	Batch functional enrichment	Yes	No	No	No	No	No	No	No
	Time-series gene expression line plot	Yes	Yes	Yes	Yes	No	No	No	No
	Heatmap plot	Yes	No	No	No	No	No	Yes	Yes
	Enrichment plot annotation	Yes	No	No	No	No	No	No	Yes
	Custom plot annotation for clusters	Yes	No	No	No	No	No	No	No
Integrated functionality comparison	Integrated clustering, enrichment, and visualization modules	Yes	No	No	No	No	No	No	No
	User-defined cluster annotation	Yes	No	No	No	No	No	No	No
	Single-cell tool integration	Yes	No	No	No	No	No	No	No
	Trajectory analysis tool integration	Yes	No	No	No	No	No	No	No
	Co-expression network analysis integration	Yes	No	No	No	No	No	No	No

*Note*: CRAN, comprehensive R archive network.

ClusterGVis is developed to simplify the complex process of data analysis and allow researchers to focus on biological insights rather than computational complexities. The tool utilizes powerful statistical methods for clustering, including fuzzy c-means clustering [9] and k-means clustering, to categorize genes or samples based on their expression profiles. Furthermore, ClusterGVis supports clustered data formats that have been pre-analyzed by other software, including Monocle2, Monocle3 [10–12], and Seurat [13] (File S1). This enables the identification of genes with similar functions or co-regulated genes under specific experimental conditions. Additionally, pathway enrichment analysis using the clusterProfiler package enables researchers to comprehend the functional roles and biological significance of different gene expression patterns. It also provides interpretation for high-throughput data, assisting researchers in understanding the biological mechanisms that underlie observed changes in gene expression.

**Figure 1:**
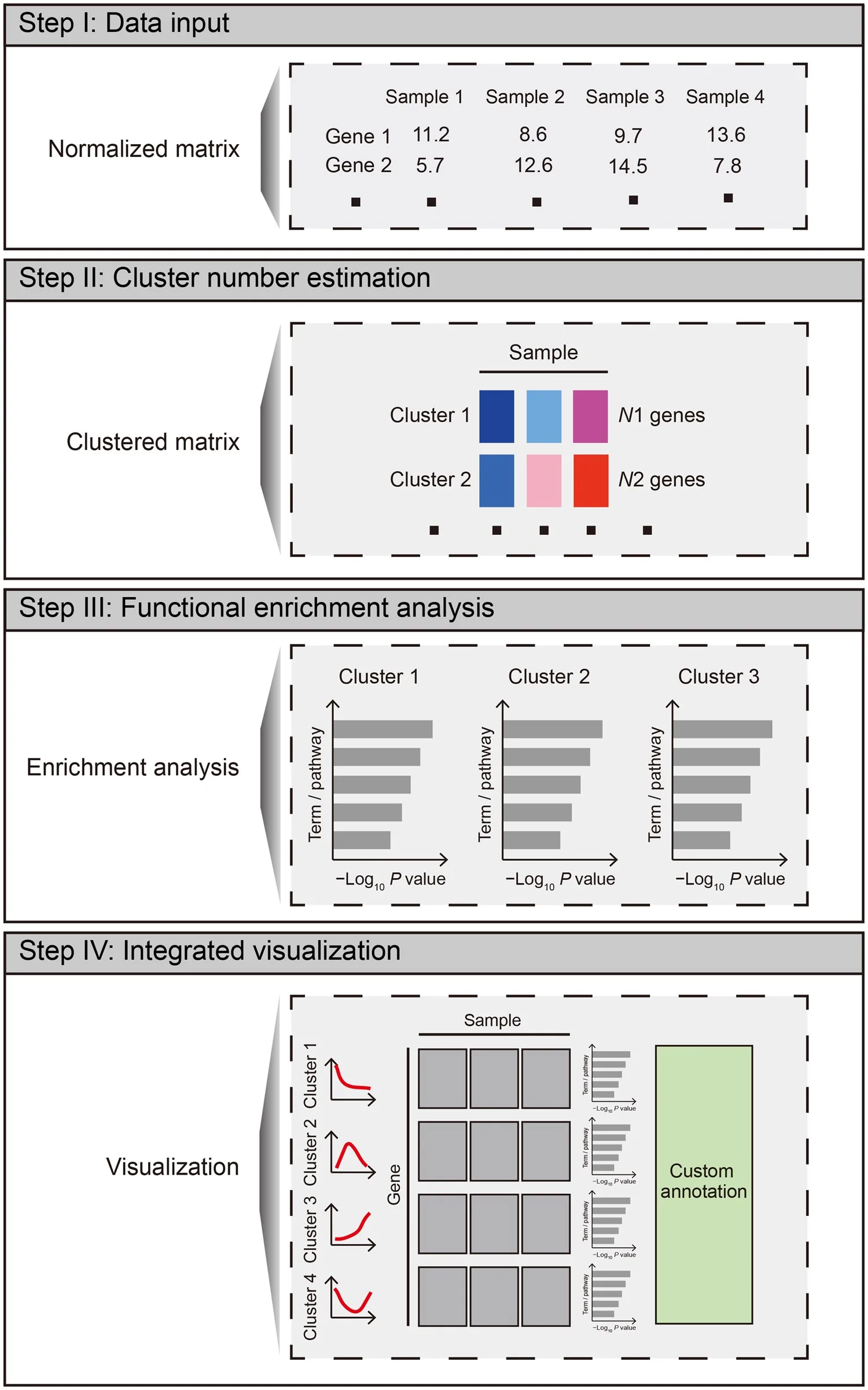
Schematic view of the ClusterGVis workflow The ClusterGVis workflow mainly includes four procedures. ClusterGVis performs k-means or fuzzy c-means clustering on a given normalized expression matrix. Then, users can perform GO or KEGG pathway enrichment analysis for each cluster. Finally, the output data from the aforementioned steps can be visualized as a comprehensive plot. GO, Gene Ontology; KEGG, Kyoto Encyclopedia of Genes and Genomes; WGCNA, weighted gene co-expression network analysis.

**Figure 2:**
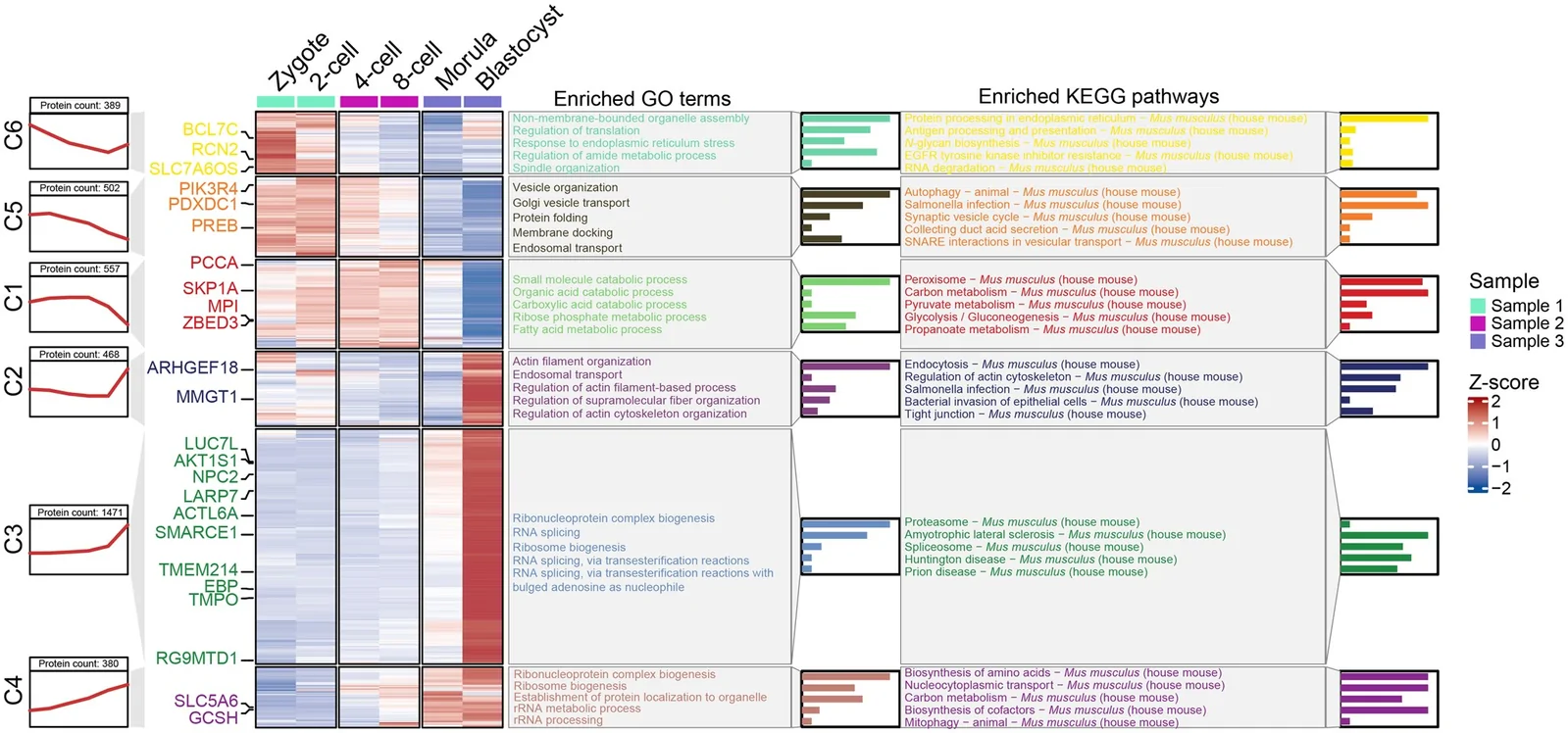
Clustered heatmap of protein expression during mouse pre-implantation development with functional enrichment annotations The line plots on the left display the overall protein expression trends for each cluster, with cluster IDs (C1 to C6) labeled on the left and protein counts indicated above. The main heatmap body, next to the line plots, illustrates normalized protein expression levels (Z-scores) across clusters, samples, and pre-implantation developmental stages. The annotations and bar plots on the right present the enriched GO terms and KEGG pathways for each cluster.

ClusterGVis is distinguished by its powerful visualization capabilities, which are crucial for interpreting gene expression patterns. The tool generates a comprehensive heatmap, offering a clear and concise overview that facilitates the identification of clusters and outliers (**Figure 1**). To demonstrate these capabilities, we applied ClusterGVis to visualize protein expression dynamics during pre-implantation development in mouse embryos [14]. The resulting heatmap clearly delineates clusters of proteins with distinct expression trends (**Figure 2**). The interactive nature of ClusterGVis allows users to delve into their data at various levels, from a comprehensive analysis to a detailed exploration of individual genes or conditions. This streamlined approach to gene expression analysis accelerates data interpretation and boosts overall research efficiency. Furthermore, ClusterGVis is adaptable to diverse gene expression datasets, making it a convenient tool for researchers across various biological fields.

In this study, we present the features and functionalities of ClusterGVis. It serves as a valuable tool for researchers exploring gene expression patterns and their implications in biological systems.

## Implementation

ClusterGVis consists primarily of four key functions: getClusters, clusterData, enrichCluster, and visCluster. These functions are designed for data preprocessing and visualization. The workflow mainly consists of four procedures (Figure 1). Figure 2 presents the comprehensive visualization achieved through these steps.

The getClusters function uses the sum-of-squared-errors (SSE) method to help users determine the optimal number of clusters. It enables users to actively redefine the number of clusters based on the visualization results, ensuring direct user involvement in the process.

**Figure 3:**
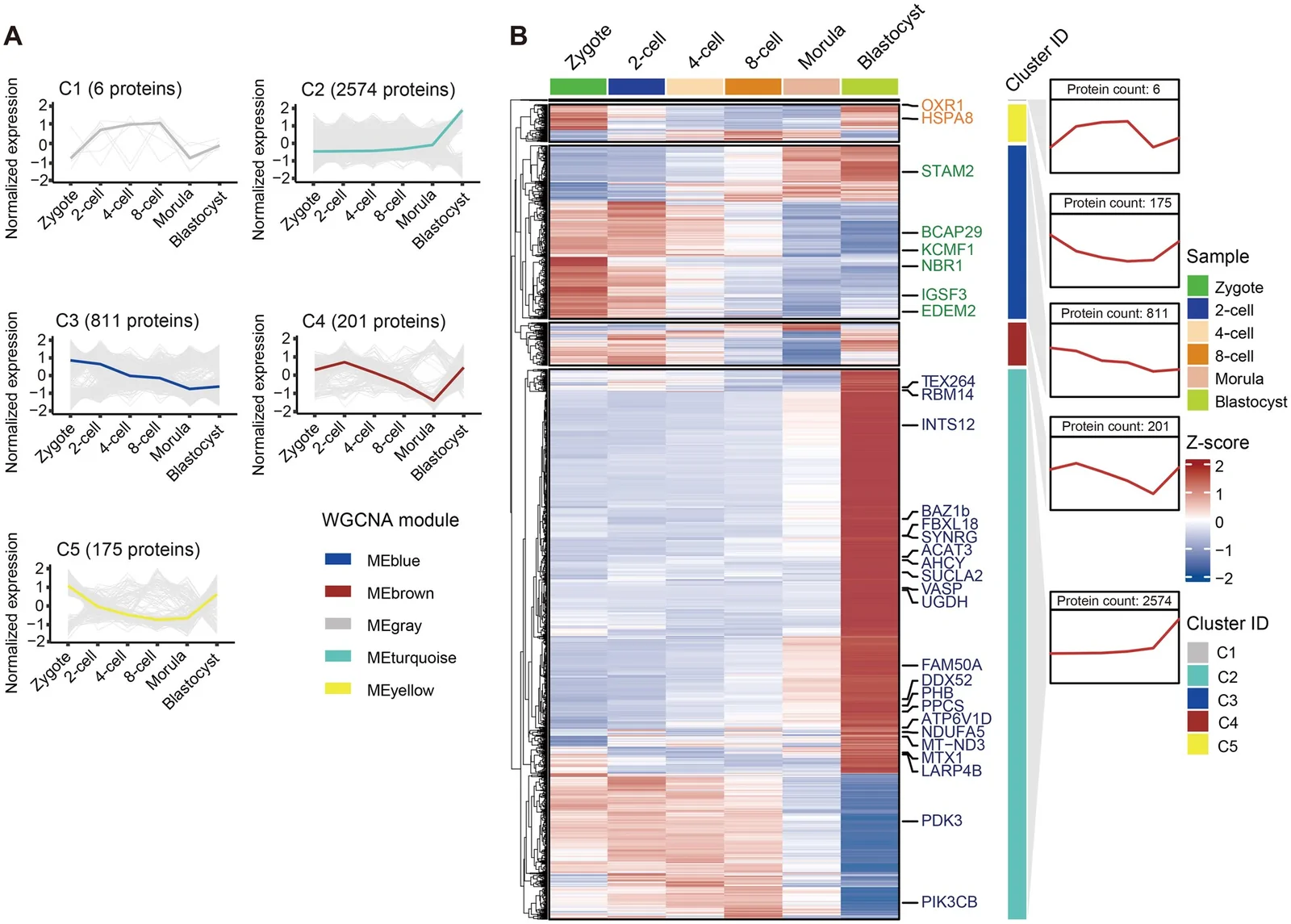
Visualization of WGCNA output using ClusterGVis **A**. Line plots showing protein expression trends of different WGCNA modules across mouse pre-implantation developmental stages. Cluster IDs are indicated above each line plot, with protein counts shown in parentheses. **B**. Heatmap displaying protein expression patterns within each WGCNA module. Rows represent individual proteins, and columns represent developmental time points.

The clusterData function aims to cluster gene expression data according to the user-specified number of clusters. It generates a list of results that contains long- and wide-format data frames for clustered data. Users can choose between the fuzzy c-means and k-means clustering methods for data clustering. Additionally, the function can handle the output object of a weighted gene co-expression network analysis (WGCNA) [15] to extract clustered data and convert them into a data frame for further analysis (**Figure 3**).

The enrichCluster function performs enrichment analysis for each cluster generated from the clusterData output. This includes analyses such as Gene Ontology (GO) enrichment, Kyoto Encyclopedia of Genes and Genomes (KEGG) pathway enrichment, and custom pathway enrichment based on user-provided gene sets. The analysis primarily utilizes the enrichR [4] and clusterProfiler [6,7] packages to perform pathway enrichment. The enrichCluster function performs pathway enrichment analysis by iteratively applying the enrichGO, enrichKEGG, or enricher functions from the clusterProfiler package to each identified gene cluster, based on a user-defined *P* value cutoff. The enrichCluster function also enables custom pathway enrichment analysis by allowing users to input their own gene-to-pathway mapping data, making it applicable to both model and non-model organisms. This adaptability increases its utility for a wide range of biological studies. The enrichCluster function outputs a data frame containing enriched pathways for each cluster. Additionally, the output from clusterData can be directly integrated with the compareCluster function from the clusterProfiler package, enabling users to seamlessly perform pathway enrichment analysis with alternative enrichment methods. The custom enrichment data frame format can be accepted directly by skipping the enrichCluster processing step.

**Figure 4:**
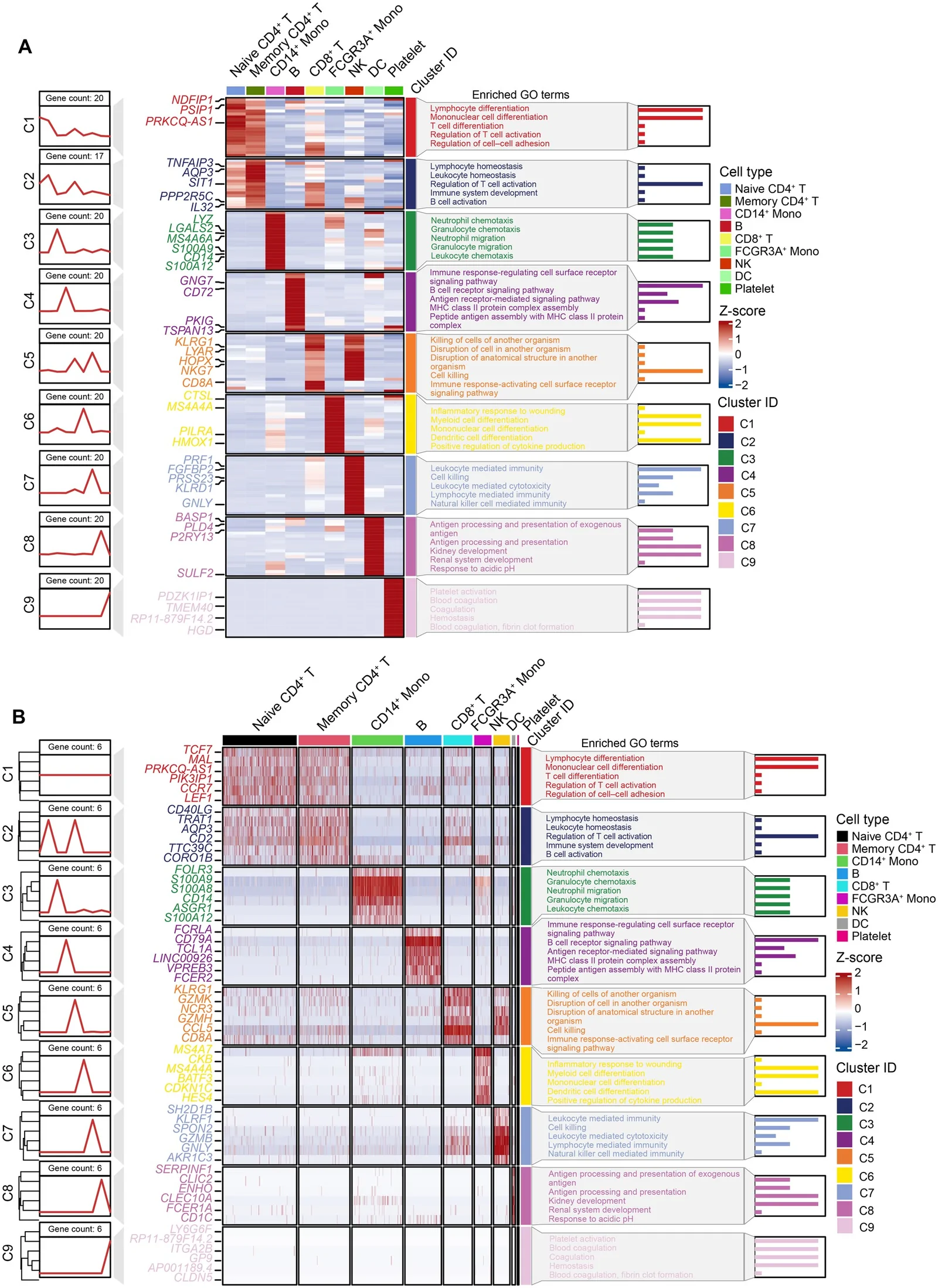
Single-cell marker gene visualization using ClusterGVis Marker gene expression patterns from the PBMC 3k dataset (the SeuratData package) are visualized at two levels: averaged expression across different cell types (**A**) and individual single-cell expression to highlight cellular heterogeneity within each cluster (**B**). PBMC, peripheral blood mononuclear cell; Mono, monocyte; NK, natural killer cell; DC, dendritic cell.

**Figure 5:**
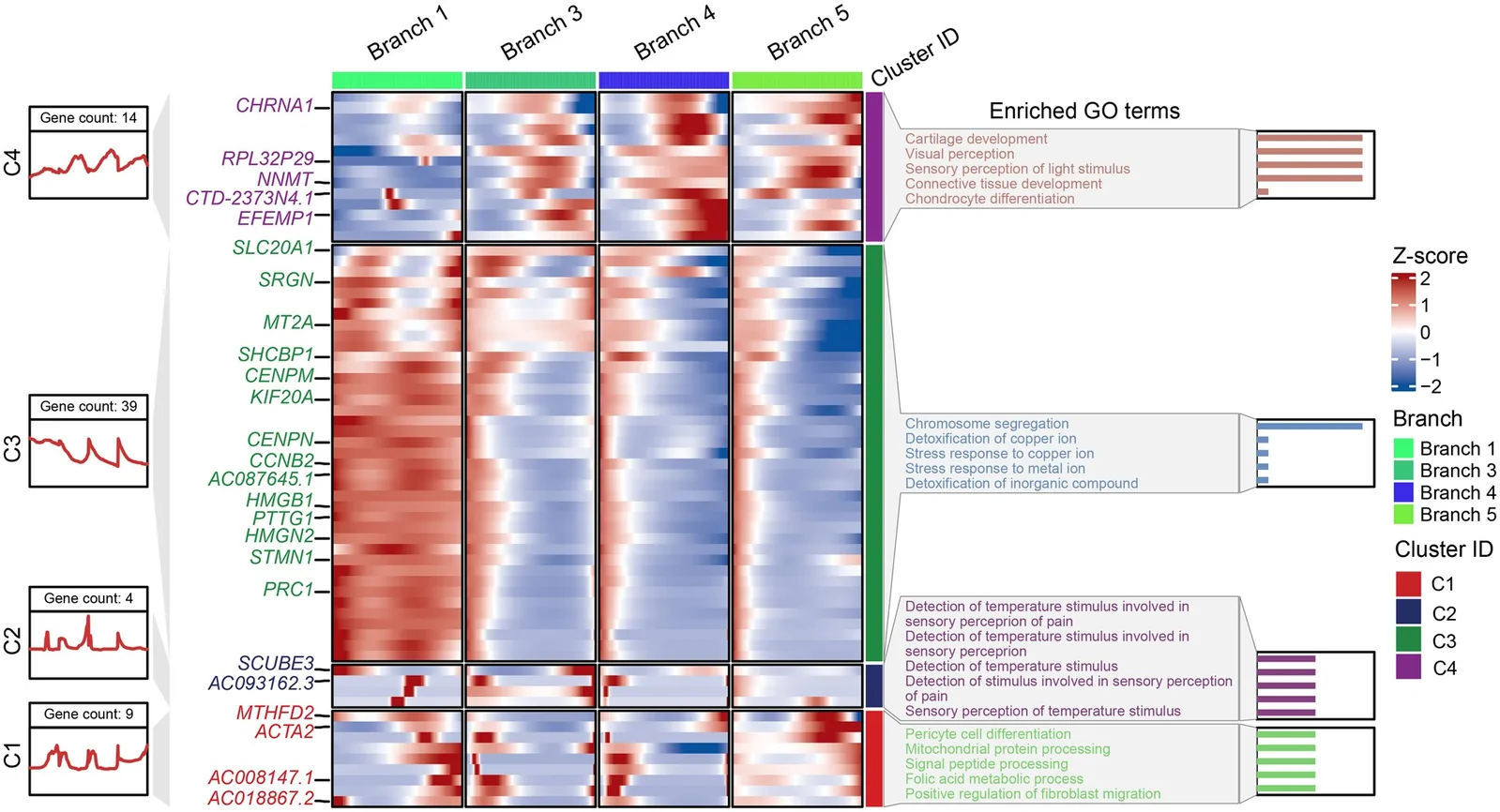
Visualization of Monocle results using ClusterGVis The integrated heatmap displays gene expression patterns along multiple differentiation branches identified by Monocle2 pseudotime analysis. The analysis was performed on single-cell RNA sequencing data of human skeletal muscle myoblasts from the HSMMSingleCell R package, showing transcriptional dynamics along the myogenic differentiation trajectory.

**Figure 6:**
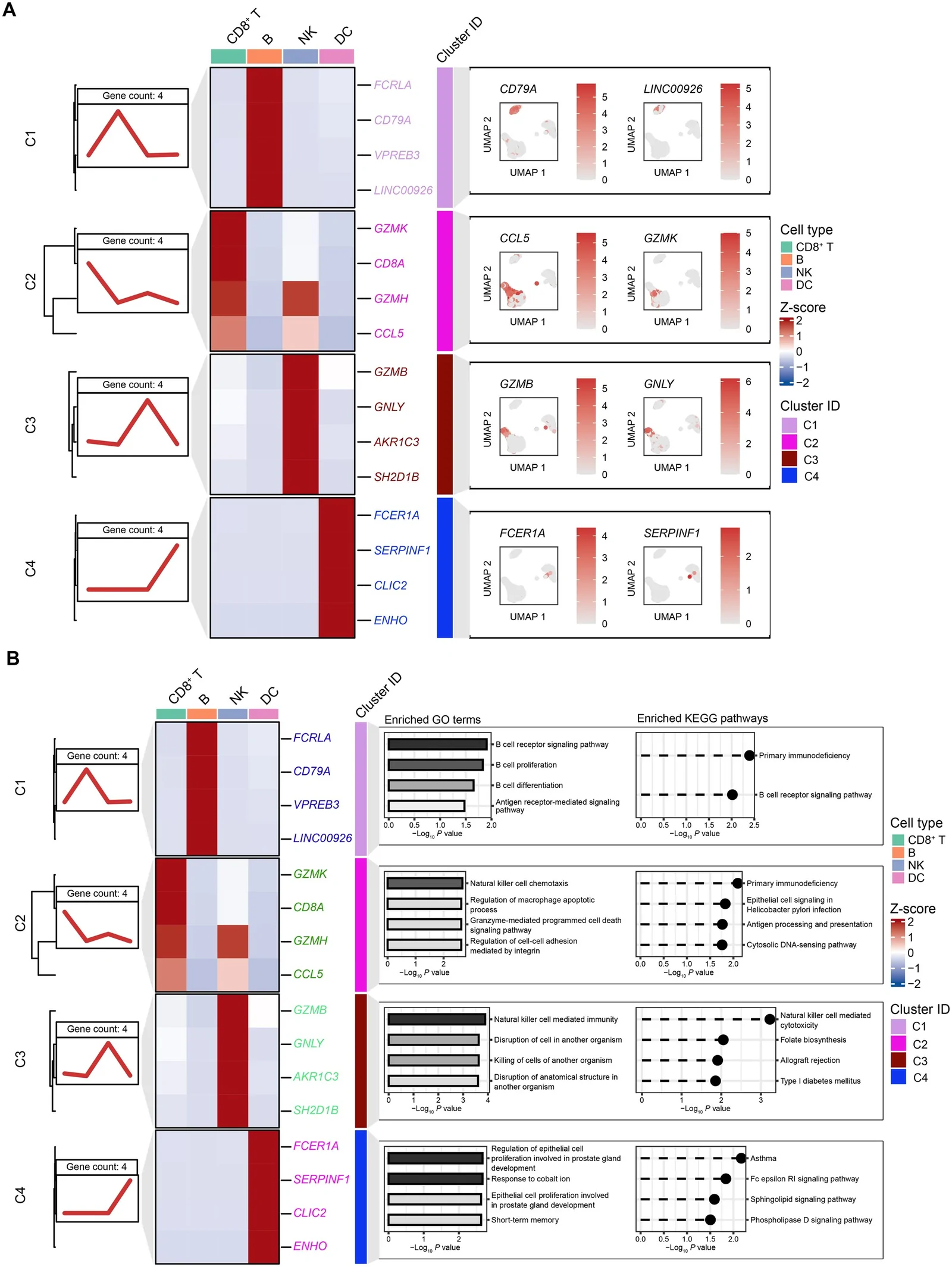
Custom insertion graphics with ClusterGVis Single-cell RNA sequencing data from the PBMC 3k dataset (the SeuratData package) are visualized with custom graphic insertions. **A**. Marker gene expression patterns across different cell types with UMAP scatter plot insertion. **B**. Marker gene expression patterns across different cell types with bar plot insertion summarizing custom GO and KEGG pathway enrichment results. UMAP, Uniform Manifold Approximation and Projection.

The visCluster function is designed to render and visualize data derived from clusterData and enrichCluster. This visualization utilizes the foundations of ggplot2 [16] and ComplexHeatmap [8], providing an integrated and comprehensive graphical representation. Additionally, it can handle outputs from the prepareDataFromscRNA function to visualize single-cell data (**Figure 4**). Pseudotime analysis using single-cell RNA sequencing data is commonly employed to examine dynamic gene regulatory programs along continuous biological processes. The visCluster function can also integrate results obtained from Monocle in various differentiation states (**Figure 5**). The function outputs a combined plot that includes a line plot, a heatmap, an enrichment pathway annotation, and a bar plot for each cluster with similar expression patterns. Furthermore, custom graphs can be added to annotate each cluster (**Figure 6**).

**Figure 7:**
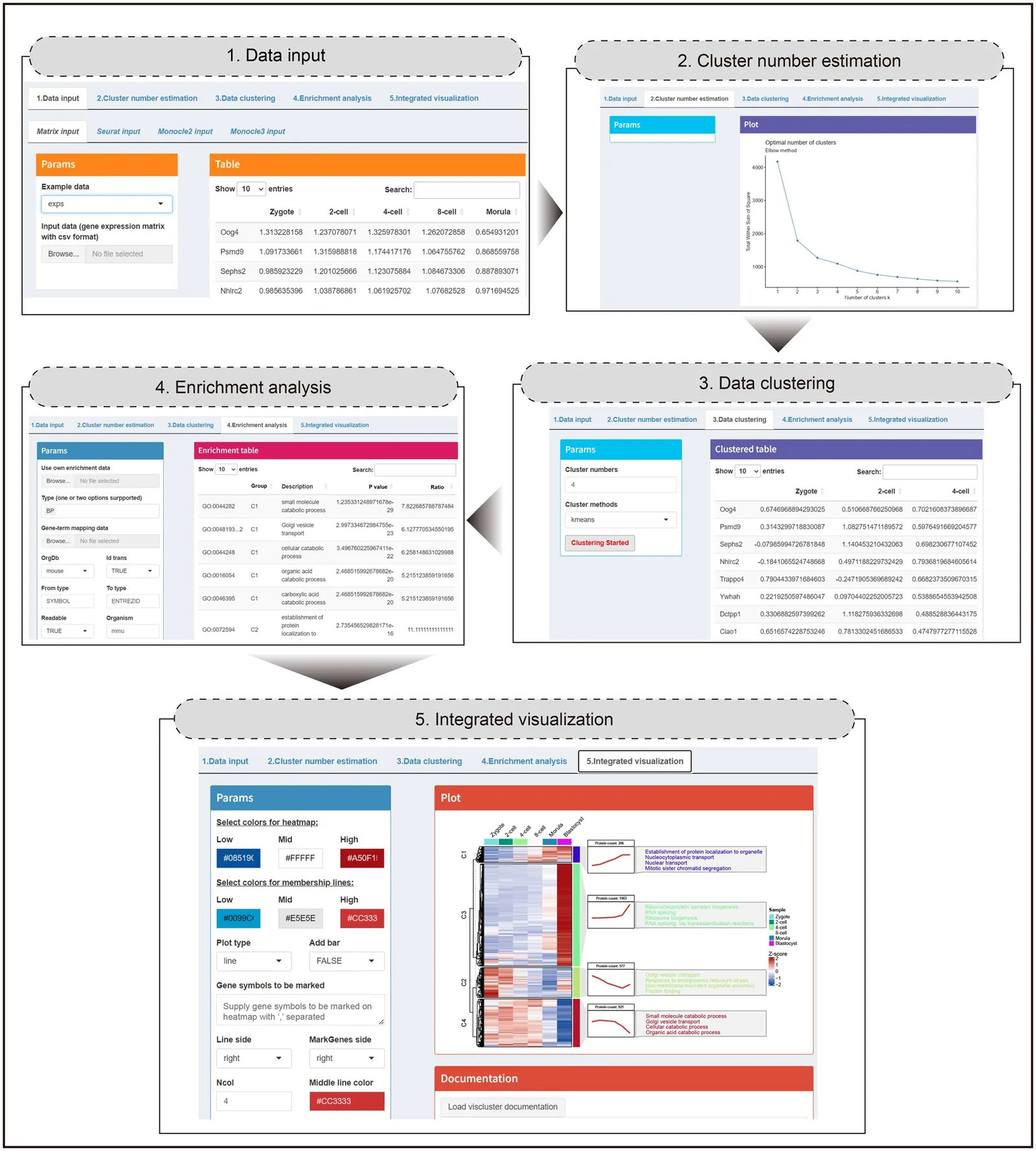
ClusterGVis Shiny app interface and workflow The application user interface consists of two main sections: a parameter control sidebar on the left and a results display panel on the right. The analysis workflow is organized into five modules: (1) Data input, (2) Cluster number estimation, (3) Data clustering, (4) Enrichment analysis, and (5) Integrated visualization. A protein expression dataset from mouse pre-implantation development [14] is used as a representative example.

In addition to developing the ClusterGVis R package for transcriptomic data analysis, we have also implemented a Shiny web application version of ClusterGVis. The Shiny app is deployed on shinyapps.io, a cloud-based service provided by Posit/RStudio. This web-based platform follows the same analytical workflows as the R package (**Figure 7**), enabling researchers without R programming experience to perform clustering, functional enrichment, and visualization tasks through an intuitive graphical interface. To further improve accessibility and flexibility, the source code of the ClusterGVis Shiny app is openly available on GitHub (https://github.com/junjunlab/ClusterGvis-app). Users can download and deploy the application locally for secure, offline data analysis. Moreover, the open-source design of the tool allows researchers to customize the source code to meet their specific analytical needs or integrate additional functionalities, promoting versatility across diverse research scenarios. With both R package and Shiny app implementations, ClusterGVis combines computational flexibility and user accessibility, enabling researchers with varying levels of technical expertise to efficiently produce publication-quality results.

## Discussion

We provide an R package, ClusterGVis, which is designed to reveal biological significance associated with multiple characteristic expression patterns. It is capable of analyzing both classic bulk RNA sequencing and single-cell RNA sequencing data. ClusterGVis allows users to generate publication-ready visualizations in R using a fundamental expression matrix as input or using output data from other software. It ensures both ease of use and the production of high-quality graphical results.

## Supplementary Material

qzag005_Supplementary_Data

## Data Availability

The processed example matrices and intermediate files generated for method evaluation and visualization demonstration in this study have been deposited in the Open Archive for Miscellaneous Data [17] at the NGDC, CNCB (OMIX: OMIX014311), and are publicly accessible at https://ngdc.cncb.ac.cn/omix.

## Code availability

ClusterGVis is an open-source R package available on GitHub (https://github.com/junjunlab/ClusterGVis) and on the Comprehensive R Archive Network (CRAN) (https://cran.r-project.org/web/packages/ClusterGVis). It has also been submitted to BioCode at the National Genomics Data Center (NGDC), China National Center for Bioinformation (CNCB) (BioCode: BT007911), which is publicly accessible at https://ngdc.cncb.ac.cn/biocode/tool/BT007911. Additionally, the ClusterGVis Shiny app is available at https://laojunjun.shinyapps.io/clustergvis_app_v0/.

## CRediT author statement

**Jun Zhang:** Conceptualization, Formal analysis, Software, Writing – original draft. **Hongyuan Li:** Formal analysis, Validation. **Wenjun Tao:** Software. **Jun Zhou:** Funding acquisition, Project administration, Supervision, Writing – review & editing. All authors have read and approved the final manuscript.
