# Supplementary material for: ClusterGVis: An Advanced Visualization and Clustering Tool for Gene Expression Analysis
Source: Genomics Proteomics Bioinformatics. 2026 Jan 22;24(2):qzag005. doi: 10.1093/gpbjnl/qzag005 (PMC13525343; doi:10.1093/gpbjnl/qzag005)
Supplement: Supplementary file 1 [file qzag005_supplementary_data.zip › qzag005_supplementary_data/File S1.docx]

**File S1 Details of ClusterGVis installation and analytical examples**

**ClusterGVis installation**

ClusterGVis is available on GitHub and Bioconductor. Users can submit issues at https://github.com/junjunlab/ClusterGVis/issues for any questions or technical support. Before installation, it is recommended to install the latest version of ComplexHeatmap from GitHub using the devtools::install_github(“jokergoo/ComplexHeatmap”) command. ClusterGVis can then be installed via GitHub [devtools::install_github(“junjunlab/ClusterGVis”)] and Bioconductor [BiocManager::install(“ClusterGVis”)].

**Example of ClusterGVis workflow**

The code required to generate all figures referenced in the main text is publicly available at https://ngdc.cncb.ac.cn/biocode/tool/BT007911. In the main text, we utilized the protein expression profiles of mouse embryos during pre-implantation development to display the analytical capabilities of ClusterGVis (**Figure 2**). The complete source code for data processing, clustering analysis, and visualization generation is available at: https://github.com/junjunlab/ClusterGVis-manuscript-code/blob/main/Figure2.R.

**Integration of WGCNA results for module visualization**

As shown in **Figures 3**A and B, ClusterGVis can directly process weighted gene co-correlation network analysis (WGCNA) output for module-based enrichment analysis and visualization. For example, users can acquire the filtered expression matrix through the network construction and module detection using WGCNA. After that, the results are further processed for visualization using the clusterData function. The complete workflow code is available at: https://github.com/junjunlab/ClusterGVis-manuscript-code/blob/main/Figure3.R.

**Seurat compatibility for single-cell data visualization**

ClusterGVis supports single-cell transcriptomic analysis results generated by Seurat. We show the integrated cluster visualizations using peripheral blood mononuclear cell (PBMC) data from the SeuratData package (**Figure 4**A and B). The entire analytical code is available at: https://github.com/junjunlab/ClusterGVis-manuscript-code/blob/main/Figure4.R.

**Pseudotime trajectory analysis with Monocle integration**

Pseudotime analysis reveals cellular differentiation trajectories and provides temporal gene expression dynamics during developmental transitions. ClusterGVis integrates with Monocle, a widely used trajectory inference tool, to enable comprehensive visualization of pseudotime-ordered data and identification of lineage-specific regulatory programs. Using a representative dataset preprocessed through Monocle, we demonstrate visualization of differentiation trajectories, temporally dynamic gene expression patterns, and functional enrichment of co-regulated modules (**Figure 5**).

The demonstration dataset for pseudotime analysis is derived from a well-characterized model system of primary human myoblast differentiation (GSE52529) [1]. This preprocessed single-cell dataset is available through the HSMMSingleCell R package and represents temporal gene expression changes during myogenic differentiation. Complete reproducible code is available at: https://github.com/junjunlab/ClusterGVis-manuscript-code/blob/main/Figure5.R.

**Flexible custom plot integration for cluster annotation**

Beyond standard visualization functions, ClusterGVis provides a flexible framework for incorporating user-generated plots as supplementary cluster annotations. This feature enables integration of diverse analytical outputs (**Figure 6**A and B). Code is at: https://github.com/junjunlab/ClusterGVis-manuscript-code/blob/main/Figure6.R.

**Reference**

[1] Trapnell C, Cacchiarelli D, Grimsby J, Pokharel P, Li S, Morse M, et al. The dynamics and regulators of cell fate decisions are revealed by pseudotemporal ordering of single cells. Nat Biotechnol 2014;32:381−6.
