# Supplementary material for: ClusterGVis: An Advanced Visualization and Clustering Tool for Gene Expression Analysis
Source: Genomics Proteomics Bioinformatics. 2026 Jan 22;24(2):qzag005. doi: 10.1093/gpbjnl/qzag005 (PMC13525343; doi:10.1093/gpbjnl/qzag005)
Supplement: Supplementary file 1 [file qzag005_supplementary_data.zip › qzag005_supplementary_data/supplementary material captions.docx]

**Supplementary material**

**File S1 Details of ClusterGVis installation and analytical examples**
